# The Practice of Medical Oncology in Morocco: The National Study of the Moroccan Group of Trialist in Medical Oncology (EVA-Onco)

**DOI:** 10.1155/2013/341565

**Published:** 2013-10-08

**Authors:** Saber Boutayeb, Amina Taleb, Rizlane Belbaraka, Nabil Ismaili, Narjiss Berrada, Wafa Allam, Halima Abahsain, Naoufal Mellas, Saif Afkir, Omar Mesbahi, Mohammed Ichou, Hassan Errihani

**Affiliations:** ^1^Department of Medical Oncology, National Institute of Oncology, Quartier Irfane, Hay Riad, 10080 Rabat, Morocco; ^2^King Mohammed VI, Quartier des Hopitaux, 20360 Casablanca, Morocco; ^3^CHU Marrakech, 53 Avenue Asif, 40 000 Marrakech, Morocco; ^4^Regional Cancer Center, Ville Nouvelle, 50070 Meknes, Morocco; ^5^Department of Medical Oncology, CHU Fes, Route de Sefrou, 30000 Fes, Morocco; ^6^Department of Medical Oncology, CHU Oujda, Oujda University, 60049 Oujda, Morocco; ^7^Department of Medical Oncology, Military Hospital, Quartier Irfane, 10080 Rabat, Morocco

## Abstract

*Objective*. To determine the current shortfall of medical oncologists (MOs) and the projected supply. *Background*. Morocco, the medical oncology (MO) is a relatively new specialty. Medical oncology was recognized as a separate specialty in 1994 but the real taking-off was done only since the 2000s after the creation of the chair of medical oncology in the University of Rabat. The GRIOMM (Moroccan group of trialist in medical oncology) was created in 2011 and conducted its first study, EVA-onco, concerning the practice of medical oncology in Morocco in 2011. *Design*. EVA-onco is a prospective study concerning the practice of medical oncology in Morocco in 2011. *Results*. The entire public cancer centers completed the survey. There were no missing data. The number of medical oncologist per 100000/habitants in Morocco was 0.09. The average number of new patients seen per medical oncologist was 718 patients (ranging by state from 97 to 1875). The shortfall of MOs was estimated at 26 at least in 2011 according to the national recommendations. *Conclusions*. Since 2010, a national strategy to increase the capacity of MO workforce existed. The current shortfall of MO is expected to disappear in the future.

## 1. Introduction

The cancer treatment is based on three primary disciplines: medical oncology, surgical oncology, and radiation oncology.

The medical oncologist specializes in diagnosing and treating cancer using chemotherapy, hormonal therapy, biological therapy, and targeted therapy. Also, he may coordinate treatment given by other specialists and gives supportive care [1].

The treatment of cancer needs a multidisciplinary team but the medical treatment of cancer is becoming more complicated and more specialized so there is a real need for a medical oncologist specialty [1]. 

In Morocco, the MO is a relatively new specialty. MO was recognized as a separate specialty in 1994 but the real taking-off was done only since the 2000s after the creation of the chair of medical oncology in the University of Rabat.

The AMFROM (Moroccan Association for training and research in medical oncology) was created in 2008 to assure a high-level of qualification for young oncologists and to improve the recognition of medical oncology. In the same way, the GRIOMM (Moroccan group of trialist in medical oncology) was created in 2011. Its first study is Eva-onco which is a national survey about the practice of medical oncology in the public health centers across Morocco.

## 2. Materiel and Methods

EVA-onco is a prospective study concerning the practice of medical oncology in Morocco in 2011 the following.

We used an electronic questionnaire with 46 item concerning:

- number and nature of practitioners,
- number and nature of paramedics,
- workload (number of new patients, number of new patients who received chemotherapy, and number of chemotherapy seats),
- center equipments (ct-scann, MRI, PET-scann, immunochemistry, emergency unit).

Statistical analysis: The data were collected and analyzed using the Microsoft office excel 97–2003 (Microsoft, Redmond, wash, USA). These data were used to calculate

- number of MOs/100 000 habitant in Morocco,
- number of MOs/100 000 habitant in each region,
- ratio of new patient/MOs/year,
- ratio of patient receiving chemotherapy.

## 3. Results

(i)* The Response Rate.* The entire 7 cancer centers completed the survey. All the items were completed, there were no missing data.

(ii)* Number of Medical Oncologists in Morocco.* In 2011, the AMFROM listed 28 medical oncologists in Morocco. There were more medical oncology positions in public (18) than private practices (6). Few medical oncology positions were located in military hospitals (4) (see Table 1).

The specialists in the public sector have salaried positions and 25% have also a university position.

All MOs in morocco have a general oncology practice and there is no subspecialty. 

(iii)* Number of MOs/100 000 Habitant in Morocco* 0.09/100, 000. The number of MOs per a one-hundred-thousand population was calculated using the population estimates provided by the Moroccan Government. The population of Morocco was estimated by the High Commissioner Office for Planning (HCP) to be at 30,566,000 in 2011 [2]. 

(iv)* Number of MOs/100 000 Habitant in Each Region.* The number of MOs per a one-hundred-thousand population was calculated using the population estimates for each region provided by the Moroccan Government [2]. 

Table 2 shows that there is an uneven geographic distribution of MOs in Morocco. The density in MOs in Hoceima's region is 4.8 times more than in the region of Marrakech. 

(v)* Ratio of New Patient/MOs/Year.* A total of 13 649 patients were treated in the public health cancer centers in morocco in 2011 with an average of 718 news patient per oncologist (ranging from 97 in Hoceima to 1875 in Casablanca) (see Table 3).

(vi)* Ratio of Act/Sit/Day.* Table 4 shows an important difference in activity between the major centers (Rabat and Casablanca) and the other centers. Also, the number of patients per seat of chemotherapy is very heterogeneous and range from 0.75/day in hoceima to 4.25/day in oujda.

(vii)* Multidisciplinary Staff Meeting.* Table 5 shows that the majority of centers tries to organize a multidisciplinary staff decision meeting but this activity is irregular and only a minority of the patients benefit from it.

## 4. Discussion

The cancer health care system in Morocco includes (in 2011) 7 public hospitals (4 university clinics) and 7 private centers. About 30000 new patients with cancer are expected every year for an estimated population of 30,566,000 habitants [3]. The health care financing in oncology is characterized by inequity, the medical insurance covers only 30% of the population. But demand for medical oncology services is expected to rise rapidly, driven by a medical assistance plan launched in 2012 for the benefit of the low-income populations in aim to give them a free access to all health care services provided by the public hospitals. Also, recently, a very active Moroccan NGO (The Foundation of Lalla Salma against cancer) is provideing anticancer drugs to all government-run oncology centers [3].

The calculated density of MOs (MOs per 100 000) in Morocco is 0.09/100 000 habitant and it is still low compared to the European and North-American countries [4–6]. The average of 718 new patients per oncologist is very high compared with the national recommendations as defined in the cancer plan against cancer (1 medical oncologist for 300 new cases). Using these recommendations, the shortfall of MOs in Morocco in 2011 is about a minimum of 26 oncologists. To our knowledge, there are no international recommendations concerning the number of MOs/habitants even if such countries like United Kingdom recommend a density of 1.1 MOs/100000 habitants [7]. The pipeline of potential medical oncologists depends on the number of residents who complete training in medical oncology. Currently, there are approximately 50 residents who are in training in three university (Rabat, Fes and Casablanca) so the current shortfall of MO is expected to disappear in the future.

Our review of the literature in Medline showed a poor literature concerning medical oncology workforce worldwide. The only published data concerned the USA, Europe, and Australia. In most emerging countries, there are large gaps in the description of oncology structure, resources, and process. Tanzania is a low-income country with a population of 42.5 million people and an estimated 21,180 new cancers in 2006. The Tanzanian staff in oncology consists in only one medical oncologist and 4 radiotherapists for the whole country [8]. This data demonstrates the huge shortfall in human resources. In low- and middle-income countries, the availability of trained human resources is one of the major factors limiting access to cancer treatment [9]. These limited human capacities in low- and middle-income countries impact, also, the policy research and the using of quality norms.

## 5. Conclusion

The data provided by Eva-onco survey could be used by the Moroccan health authorities aiming to reduce the inequalities between regions and centers concerning medical oncology workforce. 

## Figures and Tables

**Table 1:** The repartition of medical oncologists in morocco.

Region	Global	Public practice	Military hospital	Private practice
Rabat	13	5	4	4
Casablanca	4	2	—	2
Fes	2	2	—	0
Marrakech	1	1	—	0
Oujda	3	3	—	0
Agadir	2	2	—	0
Hoceima	3	3	—	0

**Table 2:** Number of MOs/100 000 habitants in each region.

Region	Number of MO's/100 000
Rabat	0.152
Casablanca	0.056
Fes	0.086
Marrakech	0.036
Oujda	0.169
Agadir	0.052
Hoceima	0.174

**Table 3:** Ratio of new patient/MOs/year.

Centre	New patient/MOs/year
Rabat	920
Casablanca	1875
Fes	333
Marrakech	1320
Oujda	426
Agadir	705
Hoceima	97
National average	718

**Table 4:** Data concerning the number of chemotherapy seats and the activity.

Centre	Number of seats	Number of chemotherapy acts	Ratio act/seat/day
Rabat	24	13143	2.14
Casablanca	28	13222	1.85
Fes	22	5154	0.77
Marrakech	13	5500	1.65
Oujda	6	6663	4.25
Agadir	22	7000	1.24
Hoceima	12	2306	0.75

**Table 5:** Data concerning the process of medical decision.

	Medical oncology staff meeting	Multidisciplinary staff decision meeting
Rabat	Yes	Yes
Casablanca	No	Yes
Fes	Yes	Yes
Marrakech	No	Yes
Oujda	Yes	Yes
Agadir	No	Yes
Hoceima	No	No

## References

[1] Warren JL, Mariotto AB, Meekins A, Topor M, Brown ML (2008). Current and future utilization of services from medical oncologists. *Journal of Clinical Oncology*.

[2] High Commissioner Office for Planning (HCP) http://www.hcp.ma/.

[3] The Lalla Salma Association against Cancer http://www.contrelecancer.ma/en/.

[4] Hortobagyi GN (2007). A shortage of oncologists? The American Society of Clinical Oncology workforce study. *Journal of Clinical Oncology*.

[5] Erikson C, Salsberg E, Forte G, Bruinooge S, Goldstein M (2007). Future supply and demand for oncologists: challenges to assuring access to oncology services. *Journal of Oncology Practice*.

[6] Blinman PL, Grimison P, Barton MB (2012). The shortage of medical oncologists: the Australian Medical Oncologist Workforce Study. *Medical Journal of Australia*.

[7] The Royal College of Radiologists (2011). *Clinical Oncology UK Workforce in 2010*.

[8] Barton MB, Frommer M, Shafiq J (2006). Role of radiotherapy in cancer control in low-income and middle-income countries. *Lancet Oncology*.

[9] Sloan FA, Gelband H (2007). *Cancer Control Opportunities in Low- and Middle-Income Countries*.

